# Metabolic-Associated Steatotic Liver Disease (MASLD): A New Term for a More Appropriate Therapy in Pediatrics?

**DOI:** 10.3390/pediatric16020025

**Published:** 2024-04-10

**Authors:** Mosca Antonella, Andrea Pietrobattista, Giuseppe Maggiore

**Affiliations:** Hepatology and Liver Transplant Unit, ERN RARE LIVER, Bambino Gesù Children’s Hospital, Istituto di ricerca, 00165 Rome, Italy; andrea.pietrobattista@opbg.net (A.P.); giuseppe.maggiore@opbg.net (G.M.)

The term “non-alcoholic fatty liver disease” (NAFLD) has been, for a long time, used to describe the spectrum of liver lesions encompassing steatosis, steatohepatitis (NASH), and steatotic cirrhosis. A form of chronic liver disease, it is prevalent worldwide, affecting more than 30% of the global population [1]. Its histological classification has been further expanded by various scoring systems concerning the degrees of steatosis, disease activity, and fibrosis [2].

In 2020, a proposal was made to change this terminology by introducing the term “metabolic dysfunction-associated fatty liver disease” (MAFLD) to replace NAFLD. While some have accepted this new terminology, concerns have been raised about the mixing of etiologies [3]. One area of particular concern was the potential negative impact of changes in diagnostic criteria for the disease in terms of biomarkers and therapeutic development. For this reason, in 2023, an expert committee used an interactive approach involving intentional sampling to generate a large global panel for this Delphi study. Based on publication records and research on fatty liver disease, the co-chairs identified 31 experts in clinical care (liver, diabetes, obesity, and nutrition), public health, and patient representation, who collectively formed the lead author group. From this consensus, it has been decided that “steatotic liver disease” remains a general term for “fatty liver disease”, encompassing the different etiologies. The term “steatohepatitis” has been deemed an important pathophysiological concept that should be retained, but “non-alcoholic steatohepatitis” (NASH) should be replaced with the term “metabolic dysfunction-associated steatohepatitis” (MASH). There was consensus to change the definition by acknowledging the presence of at least 5 out of 140 cardio-metabolic risk factors. Those with no metabolic parameters and no known cause were thought to have cryptogenic steatotic liver disease [4].

The new definitions definitively link the pathophysiology of fatty liver with metabolic dysfunction and insulin resistance, reinforcing the role of cardiometabolic risk factors associated with “metabolic dysfunction-associated steatosis liver disease”—MASLD [5].

MASLD is characterized by liver damage associated with metabolic dysfunction, lobular inflammation, ballooning, and fibrosis [6]. This condition may progress to cirrhosis and possibly to hepatocellular carcinoma (HCC). To date, a multitude of genetic, epigenetic, and environmental MASLD modifiers have been reported, especially in the perinatal period and in the first years of life. However, diagnosis is often delayed due to its non-specific clinical manifestation, with its discovery commonly occurring during routine examinations. 

MASLD, however, could lead to new scenarios for diagnosis on the pediatric level; even for children, the presence of five simple components of metabolic syndrome persists, including central or general obesity. Moreover, in this consensus, a re-evaluation of the definitions of steatohepatitis in the pediatric setting would be advantageous for clinicians’ therapeutic approach. On the other hand, the use of the term “metabolic” in the nomenclature could create confusion in the pediatric context since inborn errors of metabolism are referred to as “metabolic liver disease” [4,5].

A recent small pediatric study showed that the new MASLD criteria performed better than that for NAFLD in selecting children with obesity and higher cardio-metabolic risk, including the risk of kidney damage. Patients with MASLD showed an overall worse cardiovascular and metabolic risk profile than their counterparts with NAFLD, as supported by the presence and significant increase in HOMA-IR, uric acid, and TG/HDL-c ratio values and a higher percentage of kidney injury [7]. 

Approximately 20–30% of adult patients with MASLD develop MASH leading to liver cirrhosis and, rarely, HCC. Metabolic syndrome and its components such as obesity, impaired glucose metabolism, hypertension, and dyslipidemia are all closely associated with insulin resistance.

1 The Pathogenesis of MASLD

The pathogenesis of MASLD remains largely unknown, but, today, the fields of study are different. 

1.1 Lipogenesis: Adipose tissue is a metabolically active endocrine organ that causes the release of proinflammatory cytokines (TNF-α and Interleukin (IL)-6), whereas beneficial adipokines, such as adiponectin, become suppressed [8]. This situation leads to the development of peripheral insulin resistance with hyperinsulinemia and increased fatty acid delivery into hepatocytes. The disruption of normal insulin signaling in the hepatocyte and the increased abundance of fatty acids leads to disordered lipid metabolism with over-activation of de novo lipogenesis (DNL) transcriptional factors, causing more fatty acid and glucose products to be shunted into these lipogenetic pathways. Beta-oxidation in the mitochondria is also inhibited, as well as very-low-density lipoprotein (VLDL) packaging and export, leading to the build-up of triglycerides in the hepatocytes. Uncontrolled and incomplete lipid oxidation, oxidative stress, and protein response activation are explained as two well-characterized pathways that promote cell death in NASH [9].

1.2 Oxidative Stress: In the presence of high amounts of fatty acids in hepatocytes, oxidative stress occurs due to high levels of reactive oxygen/nitrogen species (ROS/RNS) and lipid peroxidation that are generated during the metabolism of free fatty acids in microsomes, peroxisomes, and in the mitochondria [10]. The peroxidation of plasma and intracellular membranes may cause direct cell necrosis or apoptosis, while the ROS-induced expression of Fas ligand on hepatocytes may induce cell death. 

1.3 Gut microbiota and Cytokines: An altered microbiome (“dysbiosis”) can contribute to liver damage. Human studies have documented a fecal microbiome signature characterized by increased Proteobacteria and Bacteroidetes along with a decrease in Firmicutes in patients with obesity and NASH [11]. Early studies have suggested that the intestinal microbiota is responsible for the synthesis of various hepatotoxic bacterial substances (e.g., ammonia, phenols, and ethanol). The main bacterial product involved in the pathogenesis of NASH/NAFLD is LPS, an active component of bacterial endotoxin. The endogenous production of LPS due to bacterial death induces its translocation through the intestinal capillaries thanks to a TLR4-dependent mechanism [12]. LPS, through a complex process of association with LPS-binding protein and CD14, activates TLR4 located on different inflammatory cells, increasing the expression of target genes involved in the synthesis of inflammatory cytokines such as TNF-α, IL-1, and IL-6, thus promoting insulin resistance, hepatic steatosis, hepatic inflammation, and fibrogenesis [13]. 

1.4 Genetics: Single nucleotide polymorphisms (SNPs) in the genes involved in lipid metabolism (Lipin 1—LPIN1, patatin-like phospholipase domain containing-3—PNPLA3), oxidative stress (superoxide dismutase 2—SOD2), insulin signaling (insulin receptor substrate-1—IRS-1), and fibrogenesis (Kruppel-like factor 6—KLF6) have been associated with severe NASH, but a very interesting interaction has recently been reported between genetic risk factors (PNPLA3 I148M) and the severity of steatosis and fibrosis [14]. PNPLA3 is a member of the patatin-like phospholipase family. Several studies have established that the presence of PNPLA3-GG/GC is associated with an increased risk of advanced fibrosis among patients with a variety of liver diseases and is an independent risk factor for HCC among patients with NASH [15].

2 Diagnosis

The proposed diagnostic criteria for MAFLD are based on the evidence of fatty liver disease, which can be detected by blood biomarkers, imaging techniques, or liver histology. In recent years, several non-invasive screening and diagnostic assessments have been developed for the evaluation of NAFLD.

2.1 Laboratory tests. ALT has long been accepted as an accurate indicator of liver damage and inflammation; it has been commonly used in disease monitoring and early-stage clinical trials for NAFLD. NASPGHAN recommends the assessment of ALT levels in children over 10 years of age with a BMI ≥ the 85th percentile as a screening measure for NAFLD [16]. Numerous studies have shown that the biochemical parameters of alanine aminotransferase (ALT) and aspartate aminotransferase (AST), associated with hemoglobin A1c and the homeostatic assessment model (HOMA)-IR, could provide good prediction indices of NAFLD in patients with metabolic syndrome or even in the general population [17].

Over the past 20 years, non-invasive biomarkers have been developed for the detection of steatosis and fibrosis in patients with NAFLD. In clinical practice, the NAFLD Fibrosis Score (NFS), Fibrosis-4 Index (FIB-4), and AST to Platelet Ratio Index (APRI), work well to rule out advanced fibrosis–cirrhosis and, therefore, could be used as a first-line classification to identify patients at a low risk of advanced fibrosis. However, it may be too late to take action to reverse fibrosis. Thus, the ability to detect simple steatosis and steatohepatitis early and non-invasively in patients with fatty liver is critical to preventing disease progression [18]. 

A systematic review showed that FIB-4 predicted advanced fibrosis more accurately compared to the NFS, APRI, and BARD (a score that considers the BMI, AST/ALT ratio, and diabetes). Another meta-analysis concluded that among the non-invasive simple blood scores, the NFS and FIB-4 offer the best diagnostic performance to detect advanced fibrosis in an adult population [19].

The Hepamet Fibrosis Score (HFS) was developed and validated by Ampuero et al. and is one of the most recent non-invasive fibrosis scores; it performs better than FIB-4 and NFS for the diagnosis of advanced fibrosis. The HFS was developed using data from nearly 2500 patients from five countries (Spain, France, Italy, Cuba, and China), comprising various ethnicities (Caucasian, Latino, and Asian populations) and different rates of baseline characteristics (diabetes, obesity, prevalence of fibrosis) [20]. Recently, Prasoppokakorn et al. reported the Fibrosis-8 (FIB-8) score [the FIB-4 variables plus BMI, albumin/globulin ratio, serum gamma glutamyl transpeptidase (GGT) level, and presence of T2DM] for the diagnosis of significant fibrosis. FIB-8 has been shown to have a sensitivity and specificity of 92.4% and 67.5%, respectively [21].

In the pediatric setting, the use of these scores is less frequent; a study showed that Hepamet and APRI scores perform better than the NFS and FIB-4 for the identification of significant fibrosis in patients with NAFLD but that they do not have PPVs high enough to be considered a diagnostic tool. In 173 (60.4%) adolescents presenting with fibrosis on histological analysis, the APRI and Hepamet had significant accuracy (*p* < 0.001) in distinguishing subjects with fibrosis ≥ 1, while the NFS and FIB-4 did not. The APRI had a positive predictive value (PPV) of 62.77% and Hepamet had a PPV of 63.24%. Therefore, the early detection of fibrosis in NAFLD can significantly reduce the use of liver biopsy, which is not entirely without complications in children [22].

2.2 Serum biomarkers of fibrosis. To date, we can classify specific biomarkers of fibrosis according to their molecular structure:

(1) Collagens (procollagen I and III, released in serum during deposition and remodeling of the extracellular matrix; collagen IV, released during the degradation and remodeling of the extracellular matrix).
(2) Glycoproteins and polysaccharides (hyaluronic acid, laminin, tenascin, YKL-40).
(3) Collagenases and their inhibitors; metalloproteinases (MMPs) and their inhibitors (TIMPs).
(4) Cytokines (TNF-a, IL-6, IL-8, IL-1B, and IL-10) [23].

Recently, a new panel, ELF, has been defined, which includes hyaluronic acid, type III amino terminal procollagen propeptide (PIIINP), and tissue inhibitor of metalloproteinases-1 (TIMP-1) and recommended for the determination of fibrosis [24]. ELF demonstrated good diagnostic accuracy in detecting advanced fibrosis, with a mean sensitivity and specificity of 65% and 86%, respectively, using the higher threshold of 9.8, based on a systematic review and meta-analysis by Vali et al. [25].

Hyaluronic acid (HA) is a glycosaminoglycan synthesized by hepatic stellate cells and degraded by hepatic sinusoidal cells and is a component of the extracellular matrix. In patients with chronic liver disease, especially in those with cirrhosis, its presence is an indication of the impaired functioning of hepatic sinusoidal cells and reflects an increase in the fibrogenic process.

PRO-C3, a neo-epitope-specific competitive enzyme-linked immunosorbent assay (ELISA) for PIIINP, is a relatively new direct marker of active fibrogenesis, mostly in patients with chronic hepatitis C [26]. Recent results support the use of PRO-C3 as an effective candidate biomarker for the non-invasive assessment of liver fibrosis in NAFLD. Subsequently, an algorithm called ADAPT, which incorporates PRO-C3 along with age, the presence of diabetes, and platelet count, was developed to detect advanced fibrosis in a multinational retrospective study (Australia, UK, and Japan) with 431 biopsy-proven NAFLD patients. ADAPT exhibited a negative predictive value of 96.6%, which was superior to PRO-C3 as a stand-alone marker and to existing simple fibrosis scores, namely, APRI, FIB-4, and NFS [27].

In the pediatric setting, in a study of 204 children/adolescents with NAFLD, children with NASH had higher PIIINP plasma levels and APRI and FIB-4 scores than those without NASH (*p* < 0.001). However, the PIIINP levels had a much better diagnostic performance and accuracy than the APRI and FIB-4 scores in predicting the stage of liver fibrosis. The PIIINP levels correlated with the total NAFLD activity score (NAS) and its constituent components (*p* < 0.0001). The risk of NASH or F ≥ 2 fibrosis increases progressively with increasing PIIINP levels (*p* < 0.0001). For every 3.6 ng/mL increase in PIIINP levels, the likelihood of having F ≥ 2 fibrosis increased approximately 14-fold (OR 14.1, 95% CI 5.50–35.8, *p* < 0.0001) [28].

In a number of studies, it has been shown that hepatic steatosis associated with obesity is characterized by the increased production of inflammatory cytokines by hepatocytes, which, in turn, is secondary to NF-kB stimulation, with the activation of Kupffer cells and hepatic and systemic insulin resistance. The cytokines involved are capable of producing all the classic histological features of NASH, including hepatocyte necrosis/apoptosis (TNF alpha, TGF-beta), neutrophil chemotaxis (IL-8), hepatic stellate cell activation (HSC) (TNF-alpha, TGF-beta) and Mallory body formation (TGF-beta) [29].

Another marker is fibroblast growth factor 21 (FGF-21). In fact, data generated from both adult and pediatric cohorts demonstrated that the circulating FGF-21 levels were positively correlated with NAFLD and NASH [30].

In a study of 203 obese adolescents, the authors showed that FGF-21 as a biomarker could improve the accuracy of identifying obese children with dyslipidemia and insulin resistance as well as high-grade fatty liver disease [31]. FGF-21 levels can predict the onset of simple steatosis with an accuracy of 0.661. These findings suggest that FGF-21 may have a role in identifying and monitoring the onset and prognosis of patients with NAFLD [32].

In summary, all clinical scores based on simple serum biomarkers of fibrosis are inexpensive, reproducible, and have a high sensitivity to rule out advanced fibrosis. In contrast, fibrosis-specific serum biomarkers are better at identifying patients with significant fibrosis and advanced fibrosis, but are generally more expensive and not routinely available. In addition, most of the non-invasive serum biomarkers for fibrosis have been developed and validated in the secondary and tertiary settings with a considerably higher prevalence of advanced fibrosis than in the general population, thus limiting their applicability in the primary care setting.

2.3 Imaging tests. Fatty liver disease is often diagnosed incidentally via imaging checks such as abdominal ultrasound, CT scan, or magnetic resonance imaging (MRI). The most common imaging method for diagnosis is abdominal ultrasound, which is easily accessible and can demonstrate the infiltration of fat into the liver. However, when steatosis is less than 30%, the sensitivity is significantly reduced. An alternative diagnostic method is MRI, which is highly sensitive for small amounts of isolated steatosis. Magnetic resonance spectroscopy (MRS) measures proton signals as a function of their resonant frequency to separate the signal fractions of fat and water. MRS can detect small amounts of liver fat and is considered the most accurate non-invasive method of quantifying liver fat, but this modality is not readily available or convenient [33].

More recently, ultrasound-based measurements of liver stiffness can be integrated into conventional ultrasound devices such as acoustic resonance forced pulse imaging (ARFI) and shear wave elastography (SWE) or obtained using a dedicated device, most commonly VCTE, commercially available as the FibroScan. ARFI and SWE use high-frequency ultrasonic pulses to generate fine waves and require the operator to define a region of interest and obtain a series of liver stiffness measurements. A limited number of studies of SWE and ARFI in patients with NAFLD has demonstrated very good diagnostic accuracy for advanced fibrosis [34].

3 Therapy

The treatment of MAFLD, in addition to diet and exercise, is based on the use of omega-3 fatty acids. It has been demonstrated well that polyunsaturated fatty acids of the omega-3 series, eicosapentaenoic acid and DHA, improve hepatic lipid metabolism and adipose tissue function and act as anti-inflammatory agents. A randomized study conducted in a population of 60 overweight or obese children with NAFLD tested the efficacy of DHA (250 mg/day and 500 mg/day compared to placebo) on liver fat content as assessed by ultrasound. Both dosages were able to improve steatosis, triglycerides, and ALT levels, while the dosage of DHA 250 mg/day was able to improve the histological parameters of NAFLD [35].

In a randomized, double-blind, placebo-controlled trial, the effect of DHA (500 mg) with vitamin D (800 IU) was tested in obese children with NAFLD and vitamin D deficiency. The treatment with DHA and vitamin D reduced the NAS score, HSC activation, and fibrillar collagen content. In addition, triglycerides, ALT, and IR decreased after treatment [36]. We recently carried out a randomized, double-blind, placebo-controlled trial to test the efficacy and safety of a mixture of vitamin E and hydroxytyrosol, an olive oil phenol, in adolescents with NAFLD. Patients, randomized, received either two capsules combining 7.5 mg hydroxytyrosol and 10 mg vitamin E per day or a placebo. After 4 months, the children in the treated arm showed a decrease in IR, triglyceride levels, and oxidative stress and inflammation parameters [37]. A new Phase 3 trial in pediatric NASH is currently investigating obeticholic acid, and has shown a ≥stage 1 improvement in liver fibrosis compared to the control (23% vs. 12%). However, the frequent presence of itching as a side effect (51%), the increase in serum cholesterol requiring statin therapy, and increased number of hepatobiliary events seem to outweigh the benefits. The final results of this study are yet to be published at the time of writing this article (NCT02548351) [38]. All the trials conducted on pediatric NAFLD/NASH patients to date are reported in Table 1.

In a prospective study, 101 adults with biopsy-proven NASH were put on a low-calorie diet and then randomly assigned to pioglitazone for 18 months, and the results showed that 58% achieved a two-point reduction ≥ steatosis without worsening fibrosis compared to 17% for those on placebo and 51% exhibited resolution of NASH compared to 19% for those on placebo. Liraglutide and Dulaglutide have both shown efficacy in studies investigating their use. More recently, a study conducted a 72-week, double-blind, placebo-controlled, phase II study of biopsy-confirmed NASH on Semaglutide demonstrated the resolution of NASH in 36–59% of the treated subjects compared to 17% of the controls [39].

In conclusion, lifestyle modifications remain the frontline intervention. With the increasing burden of cirrhotic disease and transplantation in adulthood, additional pharmaceutical intervention could also play a future role in the management of MAFLD in some children having to use adult medications as early as adolescence.

## Figures and Tables

**Table 1:** Therapeutic trials in pediatric NAFLD (ClinicalTrials.gov search results 1 February 2024, https://classic.clinicaltrials.gov).

	NCT Number	Title	Status	Study Results	Conditions	Interventions	Locations
1	NCT01529268	Cysteamine Bitartrate Delayed-Release for the Treatment of NAFLD in Children	Completed	Has results	Non-alcoholic fatty liver disease (NAFLD)	-Drug: DR cysteamine bitartrate capsule -Other: DR cysteamine bitartrate placebo	University of California, San Diego, San Diego, California, United States
2	NCT01913470	Study of Losartan in the Treatment of NAFLD in Children	Completed	Has results	NAFLD	Drug: Losartan	Emory University/Children’s Healthcare of Atlanta, Atlanta, Georgia, United States
3	NCT00063635	Treatment of Nonalcoholic Fatty Liver Disease in Children (TONIC)	Completed	Has results	Fatty liver	-Drug: Metformin -Dietary supplement: vitamin E -Drug: matching placebo	-University of California, San Diego, San Diego, California, United States -University of California, San Francisco, San Francisco, California, United States
4	NCT02134522	The Role of Obstructive Sleep Apnea in Children With Fatty Liver Disease	Terminated	Has results	Non-alcoholic fatty liver disease	Device: continuous positive airway pressure (CPAP).	Yale University, New Haven, Connecticut, United States
5	NCT04415112	Mediterranean Diet Treatment for NAFLD	Completed	No results available	-Mediterranean diet	-Behavioral: Mediterranean diet -Behavioral: low-fat diet	Ulas Emre Akbulut, Antalya, Turkey
6	NCT02842567	Hydroxytyrosol and Vitamin E in Pediatric NASH	Completed	No results available	NAFLD	-Drug: Hydroxytyrosol plus vitamin E -Drug: placebo	-Hepatometabolic Department, Bambino Gesù Children’s Hospital, Rome, Italy
7	NCT01934777	Efficacy and Tolerance of Treatment With DHA, Choline and Vitamin E in Children With Non-alcoholic Steatohepatitis	Completed	No results available	-Fatty liver -Liver fibrosis	-Drug: Docosahexaenoic Acid plus vitamin E plus choline Drug: placebo pearls	Bambino Gesù Hospital and Research Institute, Rome, Rome, Italy, Italy
8	NCT01285362	Fish Oil and Nonalcoholic Fatty Liver Disease (NAFLD) Study	Completed	Has results	-Non-alcoholic fatty liver disease	-Drug: fish oil supplementation -Drug: placebo supplementation	-Irving Clinical Research Center (GCRC) at Columbia University Medical Center, New York, New York, United States
9	NCT02258126	Effect of Exercise on Hepatic Fat in Overweight Children	Completed	No results available	-Non-alcoholic fatty liver disease -Obesity -Metabolic syndrome	-Other: multidisciplinary intervention program	-Pediatric Endocrinology Unit of the University Hospital of Araba (HUA), Vitoria-Gasteiz, Araba, Spain
10	NCT00655018	Effect of Vitamin E on Pediatric Nonalcoholic Fatty Liver Disease (NAFLD)	Completed	No results available	-Inflammation -Fibrosis -Insulin resistance	-Dietary supplement: vitamin treatment (alpha tocopherol plus ascorbic acid) -Dietary supplement: placebo	-Dept. Of HepatoGastoEnterology and Nutrition, Liver Unit, Rome, Italy
11	NCT01547910	Effect of Supplementation of Fish Oil on Non-alcoholic Fatty Liver Disease in Children	Completed	No results available	Non-alcoholic fatty liver disease	Dietary supplement: fish oil	Children’s Memorial Health Institute, Poland
12	NCT01553500	Glucomannan Effects on Children With Non-alcoholic Fatty Liver Disease	Completed	No Results Available	-Metabolic syndrome -Non-alcoholic fatty liver disease -Insulin resistance	-Dietary supplement: glucomannan -Behavioral: lifestyle intervention	-Bambino Gesù Children’s Hospital and Research Institute, Rome, Italy
13	NCT00823277	Metabolic Syndrome and Gen-polymorphs Influence on Weightloss Among Children in Treatment for Overweight	Completed	No results available	-Childhood Obesity -NAFLD (Non-alcoholic fatty liver disease)	-Other: chronic care multidisciplinary intervention of childhood obesity	-The Children’s Obesity Clinic, Paediatric Department, University Hospital Holbaek, Region Zealand, University of Copenhagen, Holbaek, Denmark
14	NCT01556113	Genetic Effect on Omega 3 Fatty Acids for the Treatment of Fatty Liver Disease	Completed	No results available	-Non-alcoholic fatty liver disease -Steatohepatitis -Hypertriglyceridemia	-Other: Omega diet	-Yale School of Medicine, New Haven, Connecticut, United States
15	NCT02117700	Fatty Liver Disease in Obese Children	Completed	No results available	-Obesity -Non-alcoholic fatty liver disease -Cardiovascular disease	-Dietary supplement: N-acetyl cysteine 600 mg once/day -Dietary supplement: N-acetyl cysteine 600 mg twice/day -Other: placebo twice/day	-Nemours Children’s Clinic/Alfred I duPont Hospital, Jacksonville, Florida, United States
16	NCT02644239	Impact of Ketogenic Diet on Lipoproteins in Refractory Epilepsy	Unknown status	No results available	-Epilepsy -Cardiovascular disease -Non-alcoholic fatty Liver disease -Quality of life	Dietary supplement: ketogenic diet	Nagila Raquel Teixeira Damasceno, Sao Paulo, SP, Brazil
17	NCT02098317	DHA and Vitamin D in Children With Biopsy-proven NAFLD	Completed	No results available	-NAFLD -Non-alcoholic steatohepatitis (NASH)	-Drug: DHA plus vitamin D -Drug: placebo	Bambino Gesù Children Hospital, Rome, Italy
18	NCT05073588	Effect of Indo-Mediterranean Diet on Hepatic Steatosis and Fibrosis in NAFLD Children	Completed	No results available	Non-alcoholic fatty liver disease	-Other: Indo-Mediterranean diet -Other: calorie-restricted diet	-Institute of liver and biliary sciences, Delhi, India
19	NCT03883607	Elafibranor, PK and Safety in Children and Adolescents 8 to 17 Years of Age With Non Alcoholic Steatohepatitis (NASH)	Terminated	Has results	Non-alcoholic steatohepatitis	-Drug: Elafibranor 80 mg -Drug: Elafibranor 120 mg	-University of California, San Diego, California, United States -Columbia University, New York, New York, United States
20	NCT00885313	Effects of Docosahexaenoic Acid (DHA) on Children With Nonalcoholic Fatty Liver Disease (NAFLD)	Completed	No results available	-Fatty liver -Liver fibrosis -Obesity	-Drug: DHA250 -Drug: DHA500 -Drug: PLACEBO Behavioral: lifestyle intervention	Bambino Gesù Hospital and Research Institute, Rome, Italy
21	NCT04281121	Omega 3 Supplementation in Children With Non Alcoholic Fatty Liver	Completed	No results available	Fatty liver	Dietary supplement: omega 3 fatty acids	Pediatrics hospital Ain shams University, Cairo, Egypt
22	NCT02548351	Randomized Global Phase 3 Study to Evaluate the Impact on NASH With Fibrosis of Obeticholic Acid Treatment (REGENERATE)	Terminated	No results available	Non-alcoholic steatohepatitis (NASH)	Drug: Obeticholic acid Drug: placebo	The University of Alabama at Birmingham, Birmingham, Alabama, United States Digestive Health Specialists of the Southeast, Dothan, Alabama, United States

## Data Availability

Data sharing is not applicable to this article as no datasets were generated or analyzed during the current study.
